# Bioinspired Injectable Thermosensitive Gallic Acid-Conjugated Hyaluronic Acid/Pluronic Hydrogels to Prevent Postoperative Adhesion

**DOI:** 10.3390/biomimetics11090632

**Published:** 2026-09-04

**Authors:** Jeong Yun Lee, Hyun Ho Shin, Seongyeon Jo, Da Han Hyun, Ji Hyun Ryu

**Affiliations:** 1 Department of Biomedical Materials Science, Graduate School of JABA, Wonkwang University, Iksan 54538, Jeonbuk, Republic of Korea; wjddbs4058@wku.ac.kr (J.Y.L.); tlsgusgh1231@wku.ac.kr (H.H.S.); 2 R&D Center, SCL Science Inc., Seoul 07282, Republic of Korea; josy@sclscience.com; 3 Smart Convergence Materials Analysis Center, Wonkwang University, Iksan 54538, Jeonbuk, Republic of Korea; ekgks201@wku.ac.kr

**Keywords:** hyaluronic acid, gallic acid, Pluronic F127, thermosensitive hydrogel, postoperative adhesions

## Abstract

Postsurgical adhesions are common complications of abdominal and pelvic surgeries, often leading to bowel obstruction, chronic pain, and increased risks during reoperation. Although various physical barrier materials have been developed to prevent postsurgical adhesions, retaining anti-adhesive materials at surgical sites is challenging. In this study, we developed thermosensitive injectable gallic acid-conjugated hyaluronic acid (HA-GA) and Pluronic F127 (PluF) composite hydrogels to retain anti-adhesive materials and prevent postsurgical adhesion. The incorporation of HA-GA reduced the critical gelation concentration of PluF from approximately 18 to 12 wt% and decreased the gelation temperature from 28.5 °C for PluF to 25.6 °C for HA-GA (2 wt%)/PluF hydrogels with the left-shift in sol–gel curves. The G′ values increased from 10.1 ± 1.7 kPa for PluF to 24.8 ± 3.6 kPa for HA-GA (2 wt%)/PluF hydrogels at 37 °C. In addition, the HA-GA/PluF hydrogels exhibited enhanced mass retention as a function of time compared to PluF hydrogels alone. HA-GA (2 wt%)/PluF hydrogels retained 33.4 ± 3.5% of their initial mass after 7 d, whereas PluF was completely eroded within 3 d. The anti-adhesion efficacy of the HA-GA/PluF hydrogels was evaluated using a rat cecum abrasion model. Notably, the HA-GA (2 wt%)/PluF hydrogels showed reduced adhesion scores, with an adhesion extent score of 0.67 and 2.33 on postoperative day 7 and 21, respectively, compared with the untreated control group (3 and 3 on postoperative day 7 and 21). In addition, the adhesion severity scores of HA-GA (2 wt%)/PluF hydrogel groups were 0.33 and 1.33 on postoperative day 7 and 21, respectively, compared with the untreated control groups (1.67 and 2.33 on postoperative day 7 and 21). Therefore, HA-GA/PluF hydrogels provide reversible thermosensitive properties with enhanced stability and retention, supporting their potential as injectable physical barriers for postoperative adhesion prevention.

## 1. Introduction

Postoperative adhesions frequently occur following abdominal and pelvic surgical procedures and are associated with various clinical complications, such as bowel obstruction, chronic pain, tissue damage during subsequent surgeries, and severe systemic infections [1,2,3]. Postoperative adhesions are primarily initiated by inflammatory responses and tissue injury, which promote fibroblast activation and excessive extracellular matrix deposition [2,3,4]. Abnormal attachment between adjacent tissues at surgical sites may impair organ function and increase the risk of tissue damage during subsequent operations [5,6]. For the prevention of postoperative adhesion, various physical barrier materials have been developed to temporarily separate injured tissue surfaces and prevent undesired tissue-to-tissue contact during the early postoperative period [7,8]. However, conventional anti-adhesion materials frequently exhibit insufficient conformity to irregular tissue surfaces, limited immobilization at surgical sites, and rapid loss or erosion under physiological conditions [7,8,9,10]. Thus, effective physical barrier materials that remain localized at the injured site are required to improve the immobilization and retention of anti-adhesion materials and enable successful postoperative adhesion prevention.

Injectable thermosensitive hydrogels have emerged as promising anti-adhesion barriers because they remain fluidic and injectable at room temperature and subsequently undergo gelation at physiological temperature [11,12,13,14,15]. In contrast to pre-formed film- or sheet-type barriers, injectable hydrogels can conform to complex and irregular tissue surfaces, facilitating uniform coverage of injured regions [12,14,15]. Among various thermosensitive polymers, Pluronic F127 (PluF), an amphiphilic triblock copolymer composed of poly(ethylene oxide)–poly(propylene oxide)–poly(ethylene oxide), forms temperature-dependent micelle aggregates with sol-to-gel transition above the low critical solution temperature (LCST) [16,17,18,19,20]. PluF remains in a fluid state below the LCST, whereas increasing the temperature above the LCST induces closely packed micelle aggregates to form a physically crosslinked hydrogel [16,17,18,19,20]. This thermosensitive behavior enables PluF formulations to be injected at low temperatures and immobilized through gelation at body temperature. However, PluF hydrogels alone generally exhibit limited mechanical and structural stability, with rapid erosion that may restrict their ability to maintain a persistent physical barrier during the postoperative period [21,22,23]. Previous studies have investigated HA/PluF composite hydrogels to improve the limited mechanical properties and stability of PluF hydrogels alone. HA/PluF hydrogels without chemical modification of HA still exhibit limited stability [24], whereas chemically crosslinked catechol-functionalized HA/thiolated PluF systems exhibit markedly enhanced hydrogel stability [25].

Hyaluronic acid (HA), a naturally occurring polysaccharide and major component of the extracellular matrix, is biocompatible, biodegradable, hydrophilic, viscoelastic, and can form a hydrated physical barrier [16,26]. Unmodified HA also exhibits limited mechanical stability and undergoes rapid dissolution or degradation under physiological conditions [21,22]. Inspired by nature, catechol- or gallol-functionalized HA has been synthesized to enhance its mechanical properties and stability [27,28]. Phenolic compound-conjugated HAs have been extensively investigated as tissue-interactive biomaterials inspired by nature [27]. Phenolic functional groups, including catechol and gallol moieties, can interact with biological surfaces through hydrogen bonding, π-associated interactions, and oxidation-mediated interfacial coupling [27,28,29,30]. Gallol-functionalized HA (HA-GA) has been investigated for various biomedical applications, including hemostatic patches, cartilage-regenerative patches, and postoperative anti-adhesion materials [10,28,29,30]. Notably, mixtures of HA-GA and HA have exhibited improved localization and retention at injured tissue sites, with successful prevention of postoperative adhesions [10]. The cohesive properties of HA-GA enhance the elastic moduli and stability of HA-GA/HA mixtures [10,30,31]. Thus, HA-GA is a promising additive for preparing anti-adhesive materials that can be immobilized on surgical sites.

We hypothesized that physical barriers prepared by incorporating HA-GA into PluF hydrogels could enhance immobilization and retention at surgical sites, while maintaining the thermosensitive properties of PluF hydrogels. In the present study, HA-GA/PluF composite hydrogels were developed to prevent postoperative adhesions. PluF was used to provide low-temperature injectability and body-temperature-induced gelation, whereas HA-GA was incorporated to reinforce intermolecular interactions, improve structural stability, and reduce rapid hydrogel erosion. The incorporation of HA-GA not only maintained the reversible thermoresponsive behavior of PluF, but also reduced the critical gelation concentration and gelation temperature, increased the elastic modulus at 37 °C, and enhanced in vitro and in vivo stability. In addition, HA-GA/PluF hydrogels showed improved tissue immobilization and retention under continuous aqueous perfusion compared with PluF alone. Furthermore, HA-GA/PluF hydrogels showed enhanced in vitro mass retention compared with PluF alone and concentration-dependent early in vivo retention. The HA-GA/PluF hydrogels exhibited improved immobilization on the intestinal tissue and reduced the extent and severity of adhesion in a rat cecum–abdominal wall abrasion model. These injectable thermosensitive HA-GA/PluF hydrogels are expected to be useful in versatile biomedical applications, particularly for the prevention of postoperative adhesions.

## 2. Materials and Methods

### 2.1. Materials

Hyaluronic acid (HA, 200 kDa) was purchased from Lifecore (Carlsbad, CA, USA). Adipic acid dihydrazide (ADH) and Pluronic F-127 (PluF) were purchased from Sigma-Aldrich (St. Louis, MO, USA). Gallic acid (GA) and 1-ethyl-3-(3-dimethylaminopropyl)carbodiimide hydrochloride (EDC) were purchased from TCI (Tokyo, Japan). All the other chemicals were of analytical grade.

### 2.2. Synthesis of Gallic Acid-Conjugated Hyaluronic Acid (HA-GA)

HA-GA was synthesized through carbodiimide chemistry using ADH as a linker. The carboxyl groups of HA were activated by EDC and reacted with ADH to introduce hydrazide-containing linkers onto the HA backbone. Subsequently, GA was coupled to the remaining hydrazide groups through an additional EDC-mediated reaction. Briefly, HA (1 g) was dissolved in distilled deionized water (DDW; 100 mL). After complete dissolution of HA, EDC (2 g) and ADH (0.9 g) were slowly added to initiate the reaction. After 3 h, GA (0.8 g) and EDC (2 g) were gradually added to the HA solution. The pH of the solution was maintained at 5.0 during the reactions. After 12 h, the solution was dialyzed against an aqueous solution (pH 2.0) using a dialysis membrane (MWCO = 3.5 kDa) for 2 d to remove unreacted reagents, followed by additional dialysis against DDW for 4 h. The final product was collected by lyophilization. The conjugation of GA to the HA backbones was confirmed using ultraviolet–visible (UV–Vis) spectroscopy (UV-2450, Z-202201147260; Shimadzu, Kyoto, Japan) at the Core Facility of Wonkwang University (NFEC). HA-GA was dissolved in DDW at a concentration of 1 mg/mL and GA solutions were prepared as a function of concentration (0.001, 0.005, 0.01, 0.025, 0.05, and 0.1 mg/mL) for UV–Vis spectroscopic studies. All measurements were performed in triplicate.

### 2.3. Preparation of HA-GA/Pluronic F127 Composite Hydrogels (HA-GA/PluF Hydrogels)

To prepare the HA-GA/PluF hydrogels, HA-GA and PluF were dissolved in phosphate-buffered saline (PBS, pH 7.4). HA-GA (2 and 4 wt%) was dissolved in PBS (pH 7.4) solution at room temperature and PluF (24 to 56 wt%) was dissolved in PBS (pH 7.4) solution at 4 °C, respectively. After complete dissolution, HA-GA and PluF were homogeneously mixed at room temperature and 4 °C. The final concentrations of HA-GA were adjusted to 1 and 2 wt%. The final concentration of PluF ranged from 12 to 28 wt%. The final concentrations of the HA-GA/PluF hydrogels were determined using 2×-concentrated polymer solutions. Equal volumes of the two solutions were mixed to obtain final concentrations. For instance, 2 wt% HA-GA and 36 wt% PluF were mixed to prepare HA-GA/PluF hydrogels with final concentrations of 1 wt% HA-GA and 18 wt% PluF, respectively.

### 2.4. Sol–Gel Transition Behaviors of HA-GA/PluF Hydrogels

To obtain the sol–gel transition curves of the HA-GA/PluF hydrogels, a vial inversion test was performed using a refrigerated/heating circulator equipped with a water bath (JEIO Tech, Daejeon, Republic of Korea) at a temperature range between 4 and 100 °C. Briefly, HA-GA/PluF solution (1 mL) at varying concentrations was transferred into a 2 mL vial and equilibrated in the circulating water bath at 4 °C for 5 min. Next, the temperature of the water bath was gradually increased at a rate of 1 °C every 5 min. The sol–gel transition temperature of the HA-GA/PluF hydrogels was determined as the temperature at which no flow was observed for 5 min after vial inversion. A PluF hydrogel without HA-GA was used as the control. All experiments were performed in triplicate.

### 2.5. Rheological Studies of HA-GA/PluF Hydrogels

The rheological properties of the HA-GA/PluF hydrogels were evaluated using a rotational rheometer (Kinexus Lab+, Netzsch, Selb, Germany) equipped with a parallel-plate geometry (diameter: 20 mm). The elastic modulus (G′) and viscous modulus (G″) were measured at a gap distance of 0.15 mm over a frequency range of 0.1–10 Hz under temperature conditions of 4 °C and 37 °C. Temperature-sweep measurements were performed to investigate the thermosensitive behavior and gelation temperature of the HA-GA/PluF hydrogels. The elastic modulus (G′) and viscous modulus (G″) of the HA-GA/PluF hydrogels were monitored as a function of temperature ranging from 4 to 60 °C at a fixed stress of 100 Pa and a frequency of 1 Hz. The gelation temperature was determined at the crossover point between G′ and G″. In addition, frequency sweep measurements of the HA-GA/PluF hydrogels were performed at 4 °C and 37 °C. The G′ and G″ values of the HA-GA/PluF hydrogels were monitored to evaluate the changes in viscoelastic properties as a function of HA-GA concentration. Furthermore, temperature-responsive moduli recovery of the HA-GA/PluF hydrogels was investigated by monitoring the G′ and G″ values during repeated temperature cycles of 37 °C, 4 °C, 37 °C, 4 °C, and 37 °C. The final concentrations of HA-GA were adjusted to 1 and 2 wt% and those of PluF ranged from 12 to 28 wt%. A PluF (18 wt%) hydrogel without HA-GA was used as a control. All experiments were performed in triplicate.

### 2.6. Cytocompatibility of HA-GA/PluF Hydrogels

The cytocompatibility of HA-GA/PluF hydrogels was evaluated using NIH/3T3 mouse fibroblasts and a Cell Counting Kit-8 (CCK-8; Dojindo, Kumamoto, Japan) assay. NIH/3T3 cells were cultured in Dulbecco’s Modified Eagle Medium (DMEM; Welgene, Gyeongsan, Republic of Korea) supplemented with 10% heat-inactivated bovine calf serum (BCS) and 1% penicillin/streptomycin and maintained in a humidified incubator at 37 °C with 5% CO_2_. To prepare hydrogel extracts, PluF, HA-GA (1 wt%)/PluF, and HA-GA (2 wt%)/PluF hydrogels (0.5 mL each) were immersed in 2 mL of complete DMEM and incubated at 37 °C in a humidified 5% CO_2_ atmosphere for 24 h. NIH/3T3 cells were seeded in 96-well plates at a density of 1 × 10^4^ cells/well and allowed to attach for 24 h. After removing the culture medium, 200 μL of each hydrogel extract was added to the wells. For quantitative analysis of cell viability, 20 μL of CCK-8 reagent was added to each well and incubated at 37 °C for 1 h. The absorbance was measured at 450 nm using a microplate reader (iMark; Bio-Rad, Hercules, CA, USA). NIH/3T3 cells cultured in medium alone were used as controls. All measurements were performed in quintuplicate (*n* = 5).

### 2.7. In Vitro Erosion Behaviors of HA-GA/PluF Hydrogels

The in vitro stability of HA-GA/PluF hydrogels was evaluated by measuring their mass erosion behavior. Briefly, 0.5 mL of each hydrogel was placed in a 2 mL vial, followed by the addition of 1 mL of PBS (pH 7.4) preheated to 37 °C. The hydrogels were then incubated at 37 °C. The supernatant was carefully removed at predetermined time intervals, and the remaining weight of the hydrogels was measured. The mass loss ratio was calculated by comparing the remaining hydrogel weight to the initial hydrogel weight after the removal of the supernatant. The final concentration of the HA-GA ranged from 1 to 2 wt%, whereas that of PluF was fixed at 18 wt%. All experiments were performed in triplicate.

### 2.8. Adhesion Force Measurements of HA-GA/PluF Hydrogels

The tissue adhesive properties of the HA-GA/PluF hydrogels were evaluated using a universal testing machine (MCT-2150W, A&D Company, Limited, Tokyo, Japan) equipped with a 50 N load cell. PET films were cut into 1 × 5 cm^2^ strips, and fresh porcine intestinal tissues were cut into 1 × 1 cm^2^ pieces and attached to the ends of the PET films using cyanoacrylate adhesive. Subsequently, HA-GA/PluF hydrogels (0.2 mL) were applied to the intestinal tissue of one PET film, and another tissue-mounted PET film was placed over the hydrogel to form a tissue–hydrogel–tissue assembly. The final HA-GA concentrations were 1 and 2 wt%, while the PluF concentration was fixed at 18 wt%. PluF (18 wt%) without HA-GA was used as a control. The PET films were secured to the upper and lower grips of the UTM and pulled at a constant crosshead speed of 10 mm/min until the tissue surfaces were completely separated. All experiments were performed in triplicate.

### 2.9. In Vitro Assessment of Tissue Immobilization Ability of HA-GA/PluF Hydrogels

To evaluate the tissue immobilization of the HA-GA/PluF hydrogels, PluF hydrogels with a black dye and HA-GA/PluF hydrogels with different colored dyes (blue dye for the HA-GA (1 wt%)/PluF hydrogel and red dye for the HA-GA (2 wt%)/PluF hydrogel) were applied to porcine intestine tissues. The hydrogels were allowed to stabilize on the tissue surface for 10 min, after which the porcine intestine tissues were tilted to approximately 90° at 4 °C and 37 °C, respectively. The flow of the hydrogels on the tissue surface was then observed for 10 min. In addition, porcine intestine tissues were mounted on a custom-made holder, and the holder was tilted to approximately 45° after injection of the hydrogels at 37 °C. To simulate the continuous flow of physiological fluid, a syringe containing 50 mL of pH 7.4 PBS was connected to a syringe pump (KD Scientific, Holliston, MA, USA), and pH 7.4 PBS was continuously perfused at a flow rate of 5 mL/min. Tissue immobilization of the hydrogels was then evaluated under these dynamic flow conditions. Tissue immobilization was monitored for up to 60 min. Pluronic F127 (PluF) hydrogel was used as the control. All experiments were performed in triplicate.

### 2.10. Animal Studies of HA-GA/PluF Hydrogels

The animal experiments were performed in accordance with the guidelines of KNOTUS Co., Ltd. (Incheon, Republic of Korea; IACUC No. 24-HB-0122). The in vivo stability of HA-GA/PluF hydrogels was monitored using 9-week-old male rats weighing 300–400 g. The animals were divided into two experimental groups: (1) HA-GA (1 wt%)/PluF hydrogel and (2) HA-GA (2 wt%)/PluF hydrogel. The hair on both thighs of the anesthetized rats was removed, followed by disinfection with povidone-iodine and ethanol. After a skin incision using a scalpel, the HA-GA/PluF hydrogel was applied between the skin and muscle layers of both thighs. The incision sites were closed with nylon sutures and disinfected. The remaining hydrogel mass was evaluated 1 and 3 d after implantation.

The anti-adhesion efficacy of the HA-GA/PluF hydrogels was evaluated in twenty-four 9-week-old male rats weighing 300–400 g. The animals were randomly divided into four groups: (1) untreated control, (2) commercial product (Mediclore, CGBio Co., Ltd., Seongnam, Republic of Korea), (3) HA-GA (1 wt%)/PluF hydrogel, and (4) HA-GA (2 wt%)/PluF hydrogel (*n* = 6). To evaluate the anti-adhesion performance of HA-GA/PluF hydrogels, a cecum–abdominal wall abrasion model was established. Briefly, the abdominal area of each rat was shaved and disinfected with povidone-iodine and ethanol after anesthesia. Following an abdominal incision, the cecum–abdominal wall was exposed. The cecum was placed on sterile gauze, and hemorrhagic abrasion was induced by rubbing a 1 × 1 cm^2^ area 30 times using sterile gauze. In addition, a corresponding 1 × 1 cm^2^ injury was created on the opposing abdominal wall using a scalpel. An equal volume (1 mL) of the commercial product, HA-GA (1 wt%)/PluF hydrogel, or HA-GA (2 wt%)/PluF hydrogel was applied to the injured region, whereas the control group received no treatment. After treatment, the abdominal cavity was closed using nylon sutures and the surgical site was disinfected. The degree of adhesion formation was evaluated 7 and 21 d after surgery. Adhesion scores were assessed using the criteria described by Dunn et al. and Zühlke et al. based on the extent and severity of adhesions. The extent score was determined using the percentage of the injured area involved, whereas the severity score was determined based on the strength of adhesion and difficulty of tissue separation. The extent of adhesion was graded as 0 (no adhesion), 1 (1–25% adhesion of the injured area involved), 2 (25–50% adhesion of the injured area involved), or 3 (51–100% adhesion of the injured area involved) [31]. The adhesion severity was graded as 0 (no or minimal adhesions), 1 (filmy, easily separable adhesions), 2 (partially requiring sharp dissection), 3 (requiring sharp dissection with vascularization), or 4 (severe adhesions strongly attached to organs and difficult to separate without damage) [32]. Two independent clinical researchers evaluated adhesion scores in a blinded manner.

### 2.11. Statistical Analysis

Statistical analyses were performed using the GraphPad Prism 8 software (GraphPad Software, San Diego, CA, USA). Statistical differences among groups were analyzed using one-way analysis of variance (ANOVA), followed by Tukey’s post hoc test for multiple comparisons among the corresponding experimental groups. Statistical significance was set at *p* < 0.05. All experimental data are presented as the mean ± standard deviation (SD).

## 3. Results and Discussion

### 3.1. Synthesis and Characterizations of HA-GA/PluF Hydrogels

**Figure 1:**
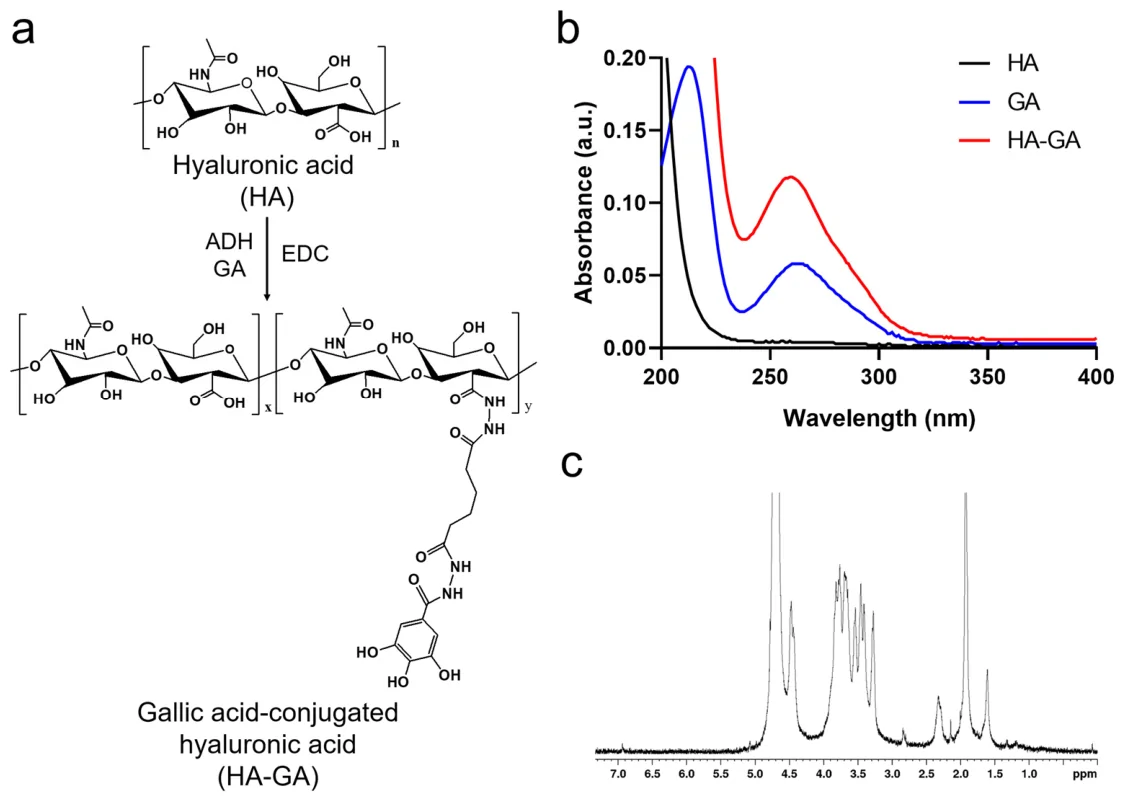
(**a**) Synthesis and chemical structure of HA-GA. (**b**) UV–Vis spectra of HA, GA, and HA-GA. (**c**) ^1^H NMR spectrum of HA-GA.

HA-GA was synthesized through carbodiimide chemistry by conjugating GA to HA using adipic ADH linkers (Figure 1a). HA was first conjugated with ADH in the presence of EDC, followed by the conjugation of GA to HA-ADH to produce HA-GA. The conjugation of GA to the HA backbone was confirmed by UV–Vis spectroscopy. In addition, UV–Vis spectroscopy was performed to confirm the conjugation of GA to HA-GA. As shown in Figure 1b, absorption at approximately 265 nm caused by GA was observed in the UV–Vis spectrum of HA-GA, which is consistent with previously reported results [10]. The degree of GA substitution in the HA backbone was measured as 3.5 ± 0.6% by comparing the absorbance of HA–gallol at 265 nm with standard curves of GA concentrations. The conjugation of GA was further confirmed by ^1^H NMR. The ^1^H NMR spectrum of HA-GA showed the characteristic methyl proton signal of acetyl groups in HA at approximately 1.9 ppm, together with aromatic proton signals attributable to the gallol moiety at approximately 6.8–7.0 ppm (Figure 1c).

### 3.2. Preparation and Sol–Gel Transition Behaviors of HA-GA/PluF Hydrogels

**Figure 2:**
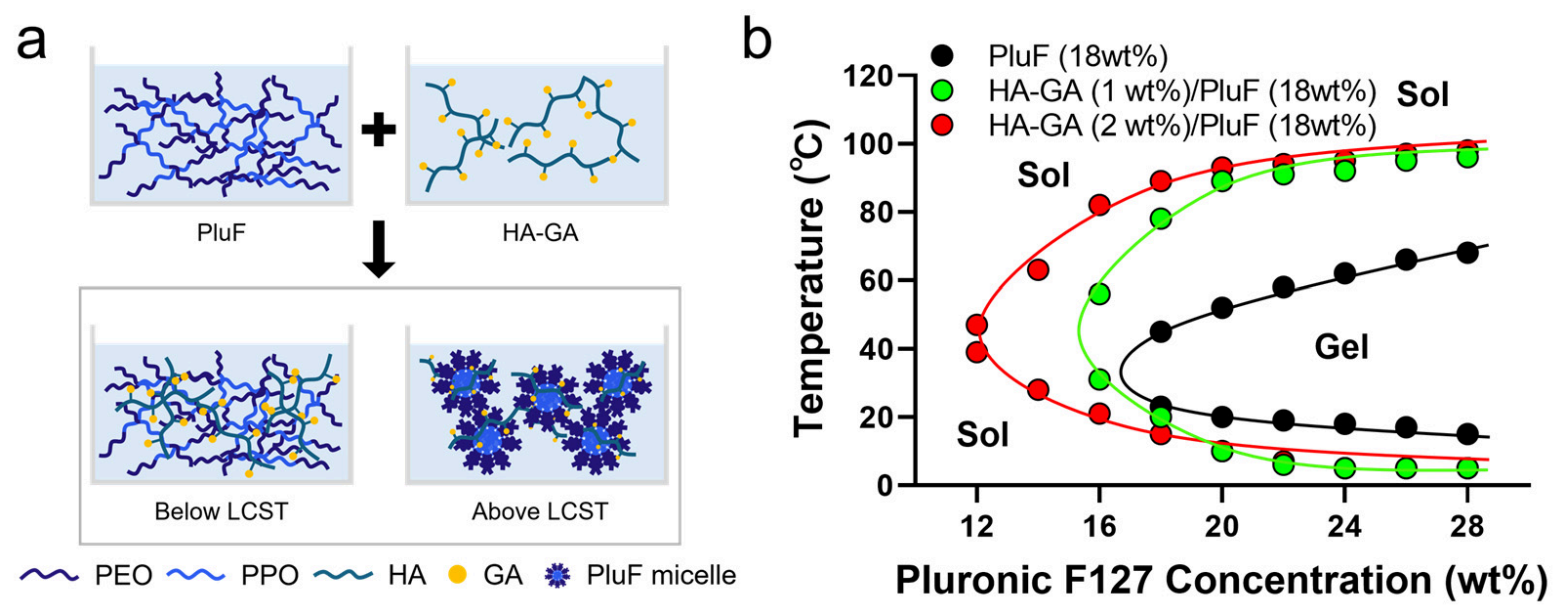
(**a**) Schematic illustration of the preparation of HA-GA/PluF hydrogels. (**b**) Sol–gel transition curves of PluF (black), HA-GA (1 wt%)/PluF (green), and HA-GA (2 wt%)/PluF (red).

As shown in Figure 2a, HA-GA and PluF solutions were homogeneously mixed in PBS (pH 7.4) to prepare HA-GA/PluF hydrogels. To investigate the effects of HA-GA concentration on the sol–gel transition behavior of HA-GA/PluF hydrogels, vial inversion tests were performed using HA-GA concentrations of 1 and 2 wt% and PluF concentrations ranging from 12 to 28 wt%. As shown in Figure 2b, the critical gelation concentration (CGC) of PluF was approximately 18 wt%. In addition, PluF hydrogels exhibited lower critical solution temperature (LCST) values of 23, 20, 19, 18, 17, and 15 °C at PluF concentrations of 18, 20, 22, 24, 26, and 28 wt%, respectively. For the HA-GA (1 wt%)/PluF hydrogel, the CGC shifted slightly to the left to approximately 16 wt%. In addition, the LCST values of the HA-GA (1 wt%)/PluF hydrogels were 31, 20, 10, 6, 5, 5, and 5 °C at PluF concentrations of 16, 18, 20, 22, 24, 26, and 28 wt%, respectively. HA-GA (2 wt%)/PluF hydrogels exhibited a left shift down to approximately 12 wt% CGC. Their LCST values were 39, 28, 21, 15, 10, 7, 5, 5, and 5 °C at PluF concentrations of 12, 14, 16, 18, 20, 22, 24, 26, and 28 wt%, respectively. As previously reported, the addition of polymers (that is, HA, chitosan, and alginate) to PluF hydrogels decreased the CGC and LCST values [24]. Therefore, the incorporation of HA-GA into the PluF hydrogels slightly affected the sol–gel transition behaviors of the CGC and LCST.

### 3.3. Temperature-Sensitive Properties of HA-GA/PluF Hydrogels

**Figure 3:**
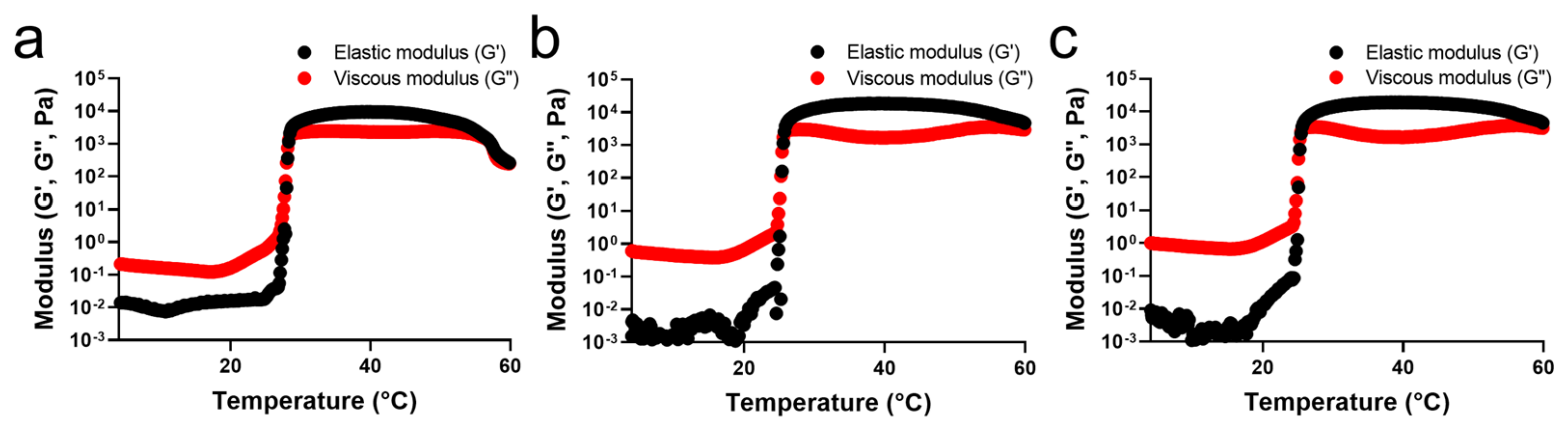
Temperature-responsive elastic modulus (G′) and viscous modulus (G″) value changes in (**a**) PluF, (**b**) HA-GA (1 wt%)/PluF, and (**c**) HA-GA (2 wt%)/PluF hydrogels.

Rheological analysis was performed to investigate the thermosensitive properties of HA-GA/PluF hydrogels. To evaluate the effects of HA-GA on the temperature-responsive behavior, the final concentration of PluF was fixed at 18 wt%, whereas the concentrations of HA-GA were adjusted to 1 and 2 wt%. The crossover points between the elastic modulus (G′) and viscous modulus (G″) were defined as the gelation temperature. Figure 3 presents the temperature-dependent changes in the elastic modulus (G′) and viscous modulus (G″) of the HA-GA/PluF and PluF hydrogels. As shown in Figure 3a, the G′ and G″ values of the PluF hydrogels crossed at approximately 28.5 °C, followed by a rapid increase in G′, indicating the typical thermosensitive sol–gel transition behavior of PluF hydrogels. In addition, both HA-GA (1 wt%)/PluF and HA-GA (2 wt%)/PluF hydrogels exhibited thermosensitive sol–gel transition behavior similar to that of PluF hydrogels. At lower temperatures (approximately 4 °C), the G″ value was higher than the G′ value in HA-GA (1 wt%)/PluF and HA-GA (2 wt%)/PluF hydrogels. The gelation temperatures were 25.7 °C for HA-GA (1 wt%)/PluF hydrogels and 25.6 °C for HA-GA (2 wt%)/PluF hydrogels (Figure 3b,c). The gelation temperature decreased with the addition of HA-GA, but no significant changes in the thermosensitive properties were observed. The incorporation of HA-GA decreased the gelation temperature while preserving the overall thermoresponsive sol–gel transition behavior. Increasing the HA-GA concentration from 1 to 2 wt% produced only a minimal additional change in gelation temperature (25.7 to 25.6 °C); both HA-GA/PluF hydrogels exhibited lower gelation temperatures than PluF alone (28.5 °C).

**Figure 4:**
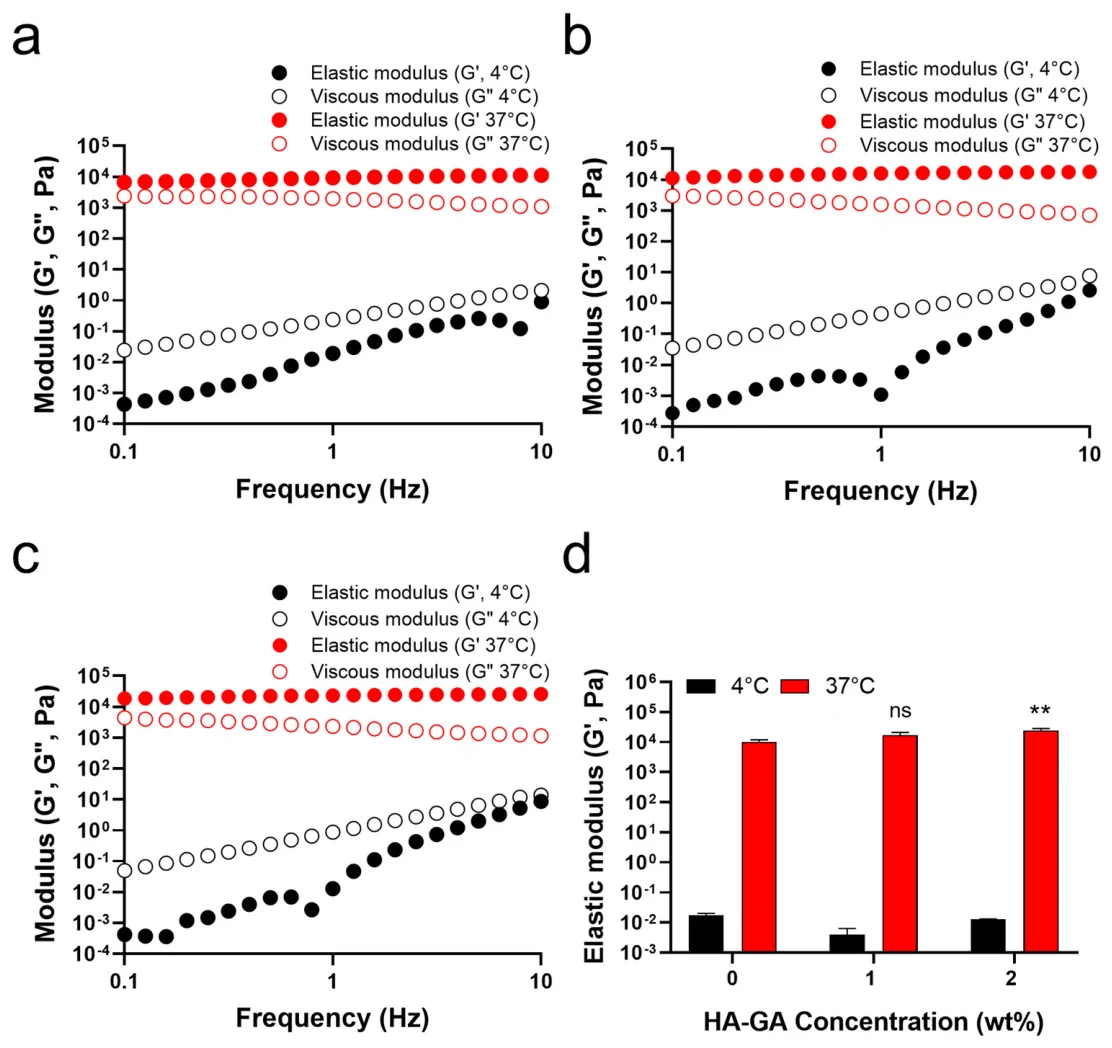
Rheological characterization of PluF and HA-GA/PluF hydrogels. (**a**–**c**) Frequency sweep measurements of (**a**) PluF hydrogels, (**b**) HA-GA (1 wt%)/PluF hydrogels, and (**c**) HA-GA (2 wt%)/PluF hydrogels. (**d**) Average elastic modulus (G′) values of PluF and HA-GA (1, 2 wt%)/PluF hydrogels at 1 Hz; (** *p* < 0.01).

The rheological properties of the hydrogels were further investigated by monitoring the G′ and G″ over a frequency range of 0.1–10 Hz at 4 and 37 °C. As shown in Figure 4a, the PluF hydrogels showed low G′ and G″ values at 4 °C, whereas G′ values were higher than G″ values at 37 °C. In addition, both HA-GA (1 wt%)/PluF and HA-GA (2 wt%)/PluF hydrogels exhibited similar rheological properties to PluF hydrogels at both 4 and 37 °C (Figure 4b,c). The average G′ values of the PluF and HA-GA (1 and 2 wt%)/PluF hydrogels were further measured at a fixed frequency of 1 Hz at 4 and 37 °C. At 4 °C, the G′ values of the PluF and HA-GA (1 and 2 wt%)/PluF hydrogels were low, approximately 20 mPa. At 37 °C, the G′ values were 10.1 ± 1.7 kPa for the PluF hydrogels, 17.0 ± 4.1 kPa for the HA-GA (1 wt%)/PluF hydrogels, and 24.8 ± 3.6 kPa for the HA-GA (2 wt%)/PluF hydrogels, respectively (Figure 4d). The corresponding G″ values were 1.95 ± 0.37 kPa for PluF hydrogels and 2.3 ± 0.5 kPa for HA-GA (2 wt%)/PluF hydrogels. Thus, the incorporation of HA-GA slightly increased the G′ values at 37 °C, enhancing the mechanical stability at physiological temperatures, as expected. In addition, the incorporation of HA-GA preserved the thermosensitive properties of PluF while increasing the G′ values at 37 °C. These data show that the PluF, HA-GA (1 wt%)/PluF, and HA-GA (2 wt%)/PluF hydrogels can be stored in 4 °C refrigerators, injected at room temperature, and form the hydrogels at 37 °C.

**Figure 5:**
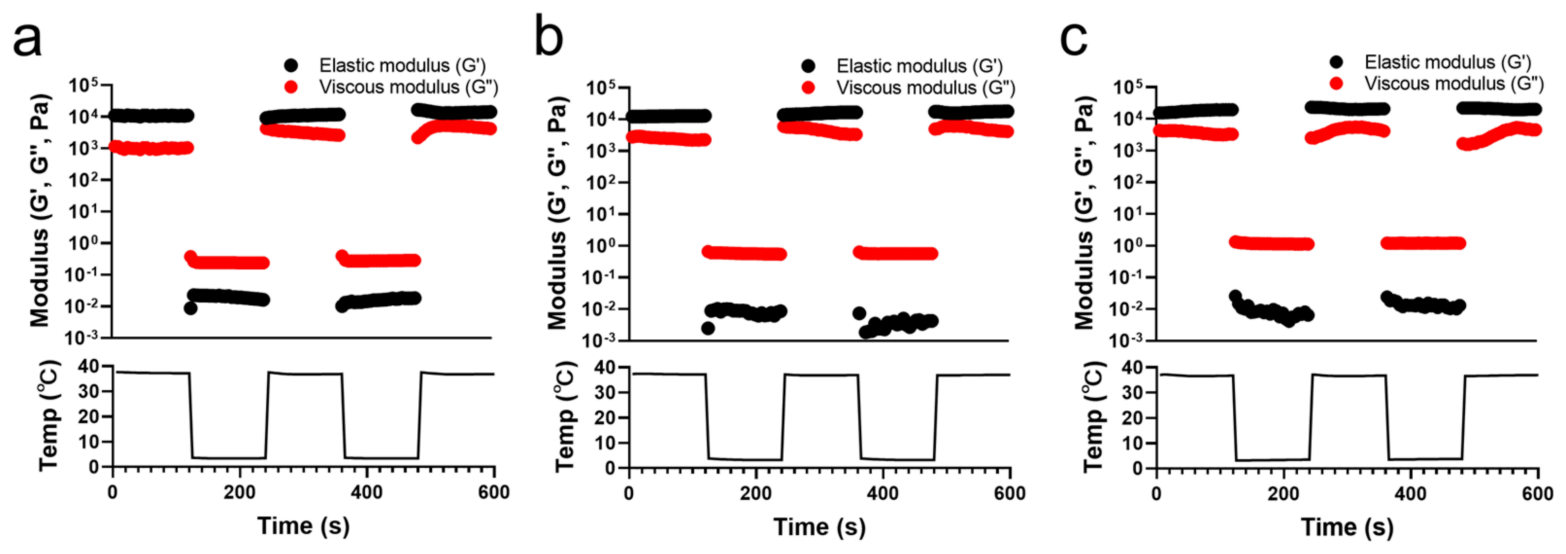
Temperature cycles of (**a**) PluF, (**b**) HA-GA (1 wt%)/PluF, and (**c**) HA-GA (2 wt%)/PluF hydrogels with repeated changes in the elastic modulus (G′) and viscous modulus (G″) during heating (37 °C) and cooling (4 °C).

To evaluate the thermoresponsive reversibility of the hydrogels, the G′ and G″ values of the PluF and HA-GA/PluF hydrogels were monitored under repeated temperature cycles, alternating between heating at 37 °C and cooling at 4 °C, with each temperature maintained for 120 s. The PluF hydrogels underwent gelation at 37 °C, returned to the sol state during the cooling cycle at 4 °C, and recovered the hydrogel networks during the heating cycle at 37 °C, exhibiting typical thermoresponsive behavior. The G′ value of the PluF hydrogels was 10.8 kPa at 37 °C and decreased to 0.02 Pa at 4 °C. Upon heating to 37 °C, the G′ value recovered to 11.0 kPa, corresponding to a recovery rate of approximately 100%. During the second cycle, the G′ value again decreased to 0.02 Pa at 4 °C and subsequently recovered to 14.1 kPa at 37 °C (Figure 5a). Along with the PluF hydrogels, the G′ values of the HA-GA (1 wt%)/PluF and HA-GA (2 wt%)/PluF hydrogels showed similar behaviors of decreased G′ values during the cooling cycle and recovered G′ values during the heating cycle (Figure 5b,c). The G′ values of the HA-GA (1 wt%)/PluF hydrogels were 12.6 kPa at 37 °C, below 0.01 Pa at 4 °C, 15.5 kPa at 37 °C (first reheating step), below 0.01 Pa at 4 °C, and 16.9 kPa at 37 °C (second reheating step), respectively (Figure 5b). The first and second recovery rates of the G′ values were 101.9% and 128.2% for PluF hydrogels, 123.0% and 109.0% for HA-GA (1 wt%)/PluF hydrogels, and 117.9% and 98.6% for HA-GA (2 wt%)/PluF hydrogels, respectively. In addition, the G′ values of the HA-GA (2 wt%)/PluF hydrogels were 17.9 kPa at 37 °C, below 0.01 Pa at 4 °C, 21.1 kPa upon the first reheating to 37 °C, below 0.01 Pa upon subsequent cooling to 4 °C, and 20.8 kPa upon the second reheating to 37 °C, corresponding to a recovery ratio of 98.5% (Figure 5c). These results also indicate that the incorporation of HA-GA into the PluF hydrogels did not impair the thermoresponsive behavior of the PluF hydrogels.

### 3.4. In Vitro Cytocompatibility of HA-GA/PluF Hydrogels

**Figure 6:**
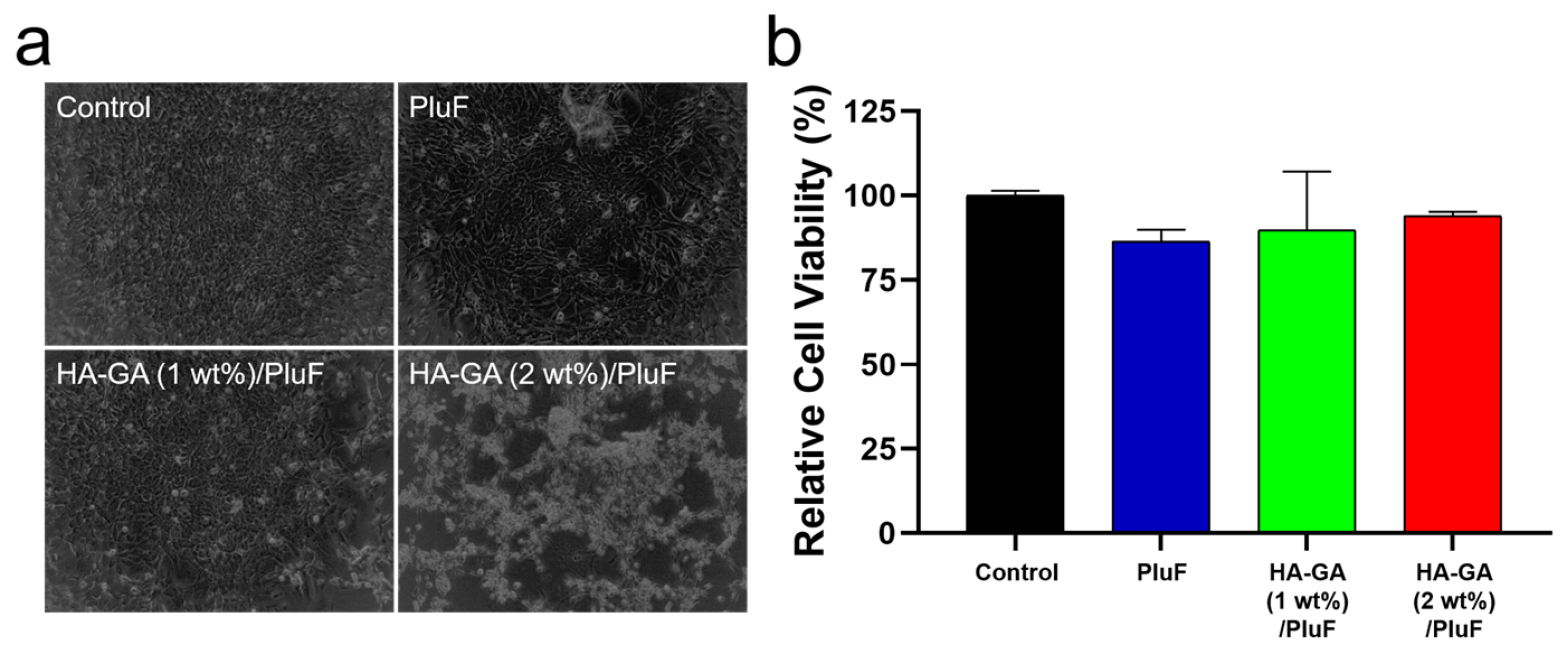
In vitro cytocompatibility of HA-GA/PluF hydrogels using hydrogel extracts. (**a**) Representative phase-contrast microscopy images of NIH/3T3 cells in the cell-only control, PluF hydrogel, HA-GA (1 wt%)/PluF hydrogel, and HA-GA (2 wt%)/PluF hydrogel groups. (**b**) Relative cell viability of NIH/3T3 cells determined by the CCK-8 assay. Data are presented as mean ± SD (*n* = 5).

The in vitro cytocompatibility of HA-GA/PluF hydrogels was evaluated using NIH/3T3 mouse fibroblasts. NIH/3T3 cells cultured in complete medium without hydrogel extract were used as the cell-only control. After 24 h of exposure to PluF, HA-GA (1 wt%)/PluF, and HA-GA (2 wt%)/PluF hydrogel extracts, the cells remained attached and maintained fibroblast-like morphology comparable to that of the control group (Figure 6a). The relative cell viability determined by the CCK-8 assay was 86.4 ± 3.4% for PluF hydrogels, 89.7 ± 17.5% for HA-GA (1 wt%)/PluF hydrogels, and 94.0 ± 1.2% for HA-GA (2 wt%)/PluF hydrogels, when normalized to the cell-only control (100%) (Figure 6b). All hydrogel extract-treated groups maintained relative cell viability above 80%, and no concentration-dependent decrease in cell viability was observed with increasing HA-GA concentrations. These results indicate that the incorporation of HA-GA did not adversely affect the cytocompatibility of the PluF hydrogels under the tested extraction conditions.

### 3.5. In Vitro and In Vivo Stability of HA-GA/PluF Hydrogels

**Figure 7:**
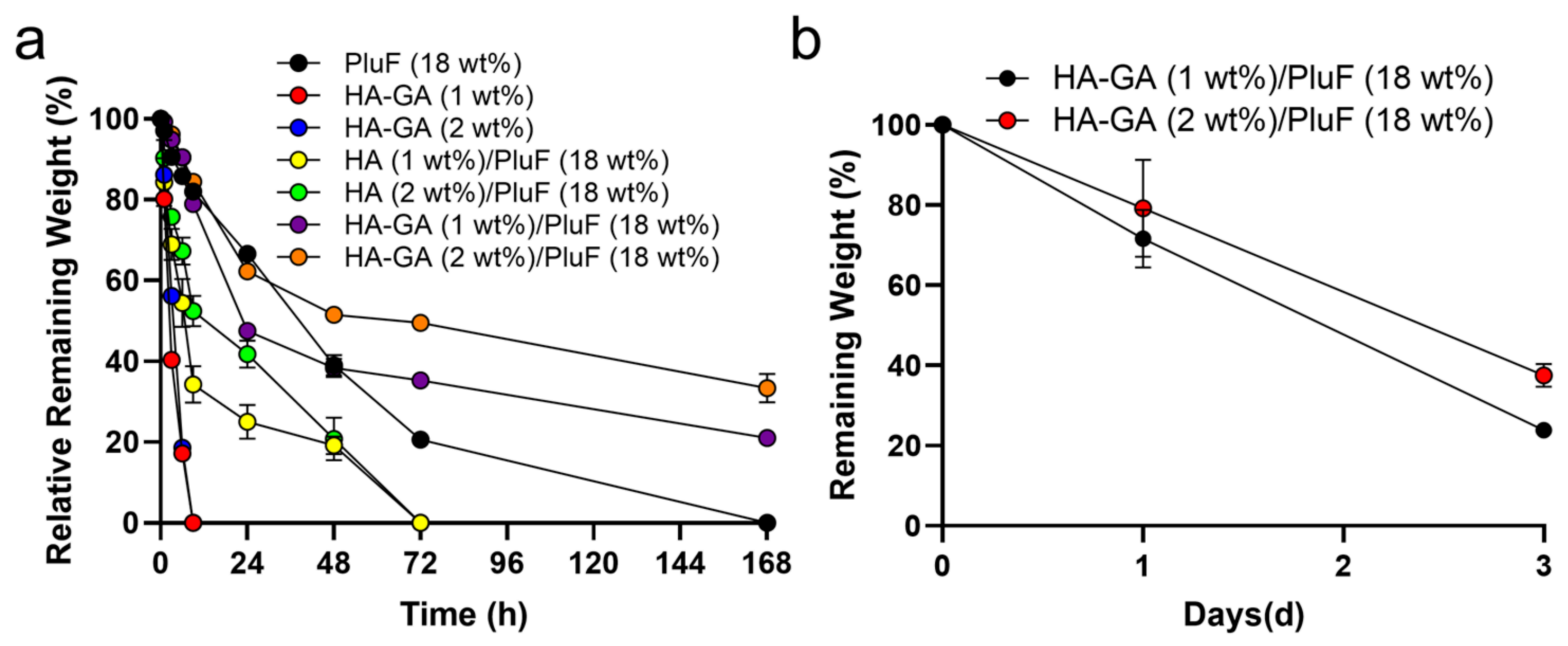
(**a**) Relative remaining weights of PluF, HA-GA (1 and 2 wt%) and HA (1 and 2 wt%)/PluF hydrogels, and HA-GA (1 and 2 wt%)/PluF hydrogels in pH 7.4 PBS solutions. (**b**) In vivo stability evaluation at 1 and 3 days after thigh incision.

The in vitro stability of HA-GA/PluF hydrogels was evaluated by monitoring the relative remaining mass of the hydrogels as a function of time. As shown in Figure 7a, uncrosslinked HA-GA solutions (1 and 2 wt%) underwent rapid dissolution and clearance because they did not form a cohesive three-dimensional hydrogel network. In addition, the PluF hydrogels rapidly eroded in PBS (pH 7.4) within 3 d, indicating their limited structural stability for anti-adhesion applications. In contrast, HA-GA (1 and 2 wt%)/PluF hydrogels remained stable for up to 7 d under physiological conditions. After 7 d, the relative remaining weights were 21.1 ± 0.7% for the HA-GA (1 wt%)/PluF hydrogels and 33.4 ± 3.5% for the HA-GA (2 wt%)/PluF hydrogels. Furthermore, HA/PluF hydrogels were completely eroded within 3 d, whereas HA-GA/PluF hydrogels remained detectable for up to 7 d. Notably, the relative remaining mass increased with increasing HA-GA concentration, revealing that the incorporation of HA-GA enhanced the structural stability of the composite hydrogels. The in vivo stability experiment was designed to evaluate early postoperative retention and was assessed at postoperative days 1 and 3. In general, postoperative adhesion formation is initiated during the early wound-healing period [33]. The remaining masses of the HA-GA (1 wt%)/PluF and HA-GA (2 wt%)/PluF hydrogels at postoperative day 1 were 71.7 ± 7.2% and 79.2 ± 12.1%, respectively. More than 70% of the HA-GA/PluF hydrogels remained on postoperative day 1. In addition, over 20% of the hydrogels remained even after 3 d under in vivo conditions (23.87 ± 0.72% for HA-GA (1 wt%)/PluF hydrogels and 37.57 ± 2.9% for HA-GA (2 wt%)/PluF hydrogels, respectively) (Figure 7b). These results reveal that increasing the HA-GA concentration enhanced the in vitro and in vivo stability of the hydrogels under physiological conditions, suggesting that HA-GA enhanced gel-state cohesion and thereby prolonged hydrogel erosion.

### 3.6. Tissue Adhesiness of HA-GA/PluF Hydrogels

**Figure 8:**
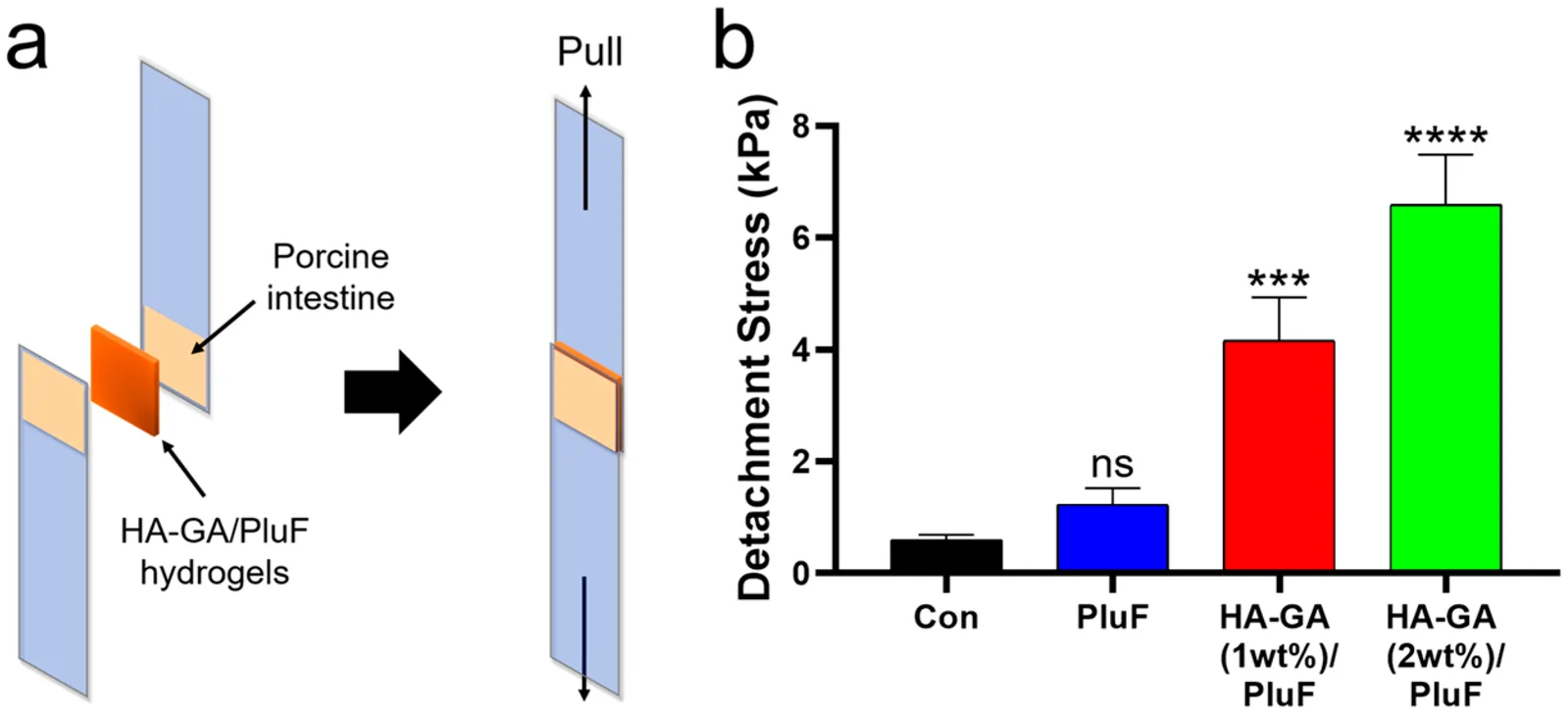
(**a**) Schematic illustration of the tissue adhesion test. (**b**) Detachment stress of PluF and HA-GA/PluF hydrogels with different HA-GA concentrations (*** *p* < 0.001, **** *p* < 0.0001).

The tissue adhesive properties of HA-GA/PluF hydrogels were evaluated using a modified lap-shear method (Figure 8a). PluF hydrogels alone exhibited a detachment stress of 1.2 ± 0.2 kPa, whereas HA-GA (1 wt%)/PluF and HA-GA (2 wt%)/PluF hydrogels showed detachment stresses of 4.2 ± 0.6 kPa and 6.6 ± 0.7 kPa, respectively (Figure 8b). This slight increase in detachment stress of the hydrogels indicated that HA-GA enhanced wet-tissue retention in physiological conditions. In addition, the tissue adhesion strength of the HA-GA/PluF hydrogels was within the range reported for adhesive hydrogels used for postsurgical adhesion prevention [10,34]. As previously reported, mussel-inspired hydrogels have been developed as a physical barrier, resulting in prevention of postsurgical adhesion [34]. In addition, fibrin-based glues have been reported to reduce postoperative adhesion by covering injured tissue surfaces and maintaining physical separation [35]. Thus, the slight tissue adhesiveness of HA-GA/PluF hydrogels may instead facilitate localization of the hydrogel barrier and help maintain physical separation between opposing injured tissues.

### 3.7. In Vitro Immobilization Ability of HA-GA/PluF Hydrogels

**Figure 9:**
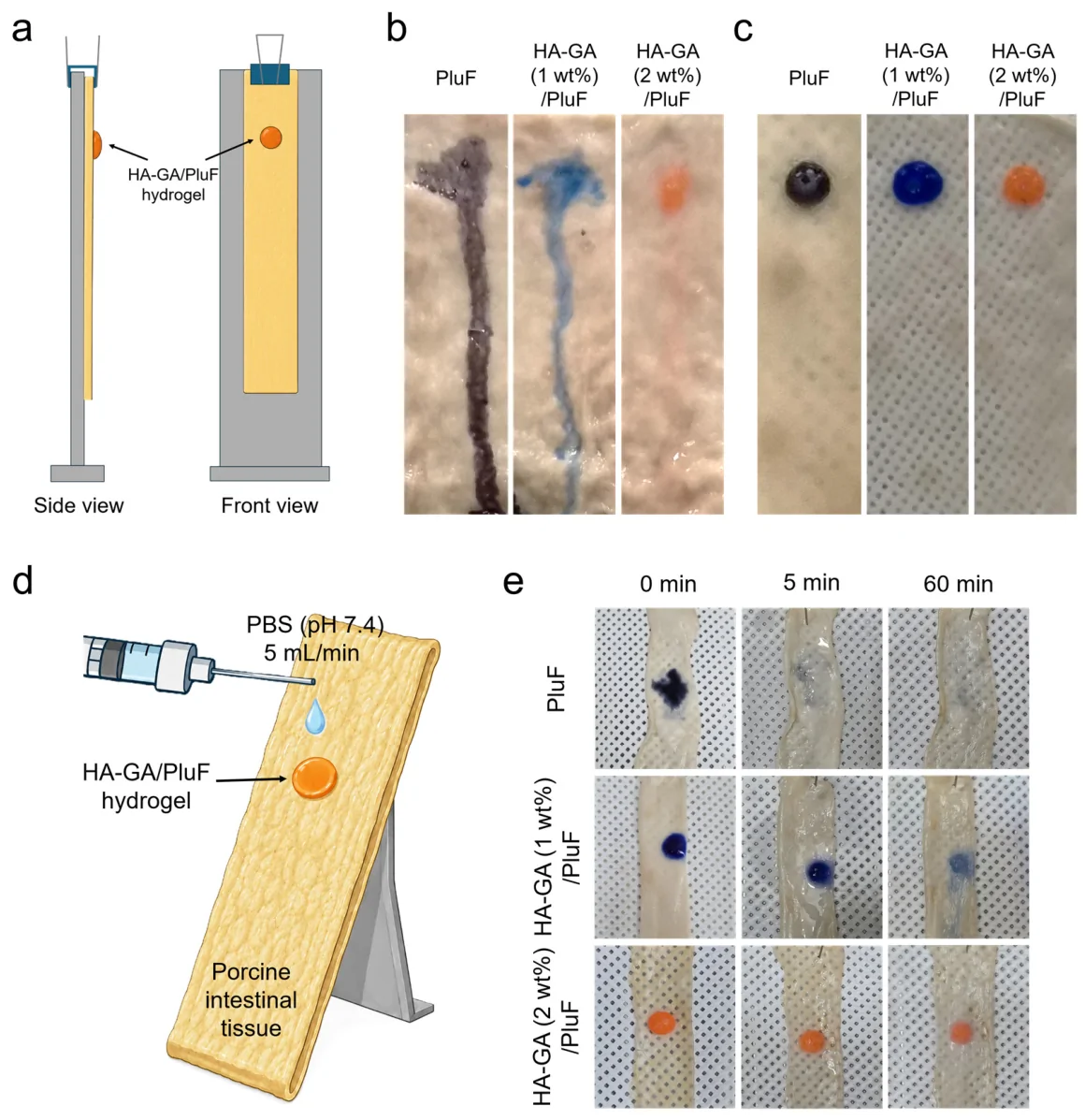
Evaluation of tissue immobilization of hydrogels for postoperative adhesion prevention. (**a**) Schematic illustration of the experimental setup for evaluating hydrogel immobilization on porcine intestinal tissue tilted at approximately 90°. (**b**) Representative images showing the flow behavior of PluF and HA-GA/PluF hydrogels on porcine intestinal tissue at 4 °C. (**c**) Representative images showing hydrogel immobilization on porcine intestinal tissue at 37 °C. (**d**) Schematic illustration of the hydrogel retention test on porcine intestinal tissue tilted at approximately 45° under continuous PBS perfusion (pH 7.4, 5 mL/min) at 37 °C. (**e**) Representative images showing the retention of PluF and HA-GA/PluF hydrogels during continuous PBS perfusion.

To evaluate the enhanced tissue immobilization and retention of the HA-GA/PluF hydrogels for postoperative adhesion prevention, in vitro tissue immobilization experiments were performed (Figure 9). As shown in Figure 9a, PluF and HA-GA (1 and 2 wt%)/PluF hydrogels were applied to porcine intestine tissues under 4 °C and 37 °C conditions, respectively. When the porcine intestine tissues were tilted to approximately 90°, the PluF and HA-GA (1 and 2 wt%)/PluF hydrogels maintained a sol state and flowed downward at 4 °C (Figure 9b). In contrast, when the porcine intestine tissues were tilted to approximately 90°, both the PluF and HA-GA (1 and 2 wt%)/PluF hydrogels remained in a gel state, and no visible flow was observed on the tissue surface at 37 °C (Figure 9c). To further evaluate the tissue retention of HA-GA/PluF hydrogels under dynamic conditions, pH 7.4 PBS was continuously supplied at a flow rate of 5 mL/min for 60 min (Figure 9d). The PluF hydrogel gradually flowed downward and was rapidly washed away, whereas the HA-GA (1 and 2 wt%)/PluF hydrogels exhibited markedly slower flow rates and visibly greater retention throughout the observation period (Figure 9e). The HA-GA (1 and 2 wt%)/PluF hydrogels demonstrated superior tissue immobilization compared with the PluF hydrogel alone. Conventional film- or sheet-type anti-adhesion barriers can effectively separate injured tissue surfaces but may show limited conformity to irregular or difficult-to-access surgical sites. In contrast, injectable thermosensitive hydrogels provide improved conformability and coverage, although insufficient retention and rapid washout may limit their barrier function. In the present study, continuous PBS perfusion was used to evaluate hydrogel retention under dynamic conditions. These results suggest that the incorporation of HA-GA effectively enhances hydrogel retention at the surgical site, thereby prolonging its residence time for postoperative adhesion prevention.

### 3.8. Prevention of Postsurgical Adhesion of HA-GA/PluF Hydrogels

**Figure 10:**
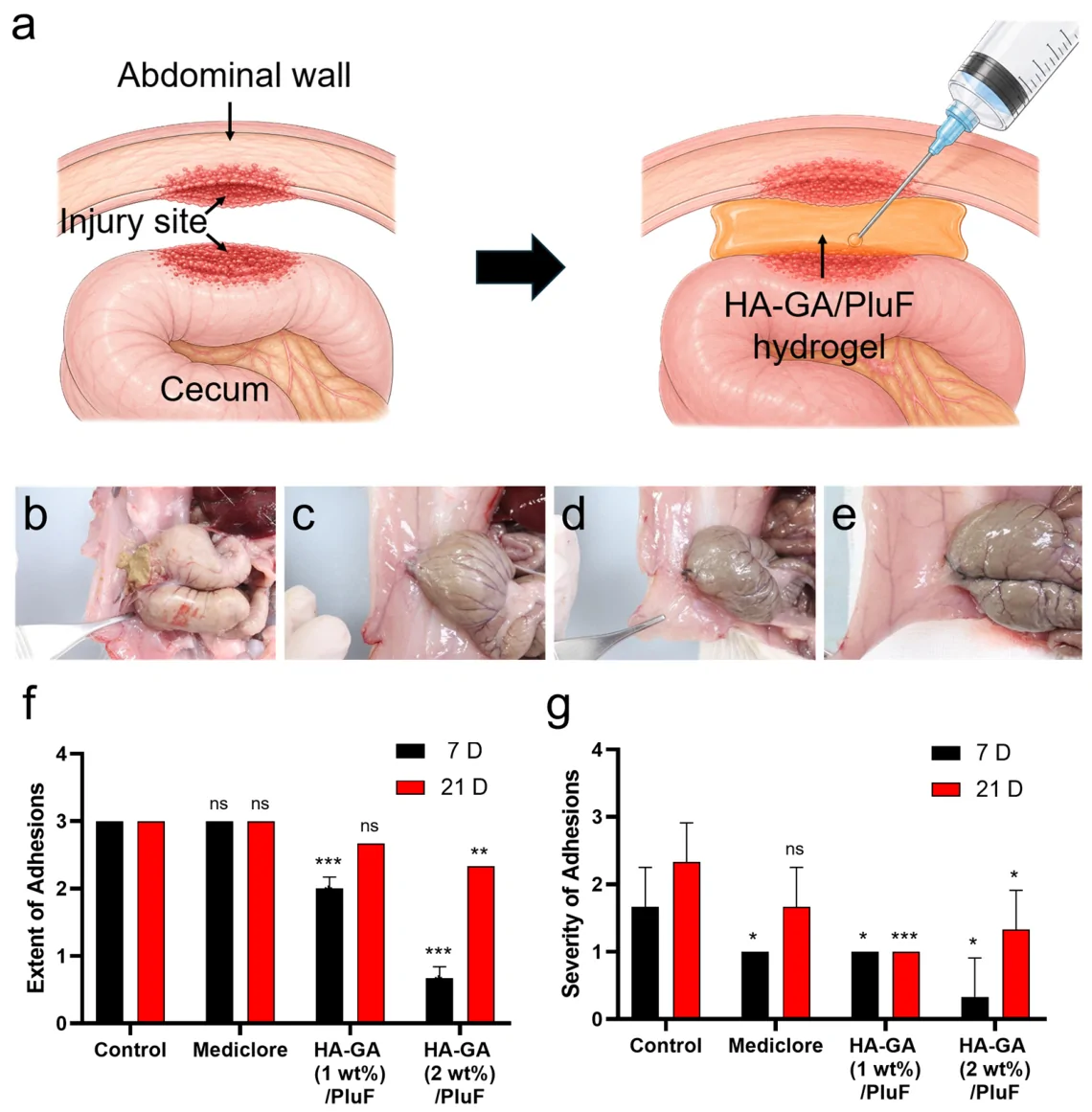
(**a**) Schematic illustration of an in vivo cecum–abdominal wall abrasion model for evaluation of postsurgical adhesion prevention. (**b**–**e**) Representative images of postsurgical adhesion sites at 21 days after surgery: (**b**) untreated control, (**c**) commercial anti-adhesive product (Mediclore), (**d**) HA-GA (1 wt%)/PluF hydrogel, and (**e**) HA-GA (2 wt%)/PluF hydrogel. (**f**) Adhesion extent scores on postoperative days 7 and 21. (**g**) Adhesion severity scores on postoperative days 7 and 21. Statistical comparisons were performed against the untreated control group within each postoperative time point (day 7 or day 21). * *p* < 0.05; ** p < 0.01; *** *p* < 0.001 vs. the corresponding untreated control.

The anti-adhesion efficacy of HA-GA/PluF hydrogels was evaluated using a rat cecum–abdominal wall abrasion model. The survival rates were 100% for all control and experimental groups throughout the experimental period. As shown in Figure 10a, abrasion injuries were induced in the cecum–abdominal wall using sterile gauze and a scalpel. Subsequently, the commercial product or HA-GA/PluF hydrogels were applied to the injured regions, and the adhesion scores were evaluated on postoperative days 7 and 21 using anti-adhesive scoring by two independent clinical researchers in a blinded manner. For the adhesion extent evaluation, the control and commercial product groups exhibited adhesion scores of 3 on postoperative day 7, whereas the HA-GA (1 wt%)/PluF and HA-GA (2 wt%)/PluF hydrogels showed lower adhesion scores of 2 and 0.67, respectively (Figure 10f). On postoperative day 21, the adhesion scores were 3 for the control group, 3 for the commercial product group, 2.67 for the HA-GA (1 wt%)/PluF hydrogels, and 2.33 for the HA-GA (2 wt%)/PluF hydrogels. In the evaluation of adhesion severity, the control and commercial product groups showed adhesion scores of 1.67 and 1 at postoperative day 7, respectively, whereas HA-GA (1 wt%)/PluF and HA-GA (2 wt%)/PluF hydrogels exhibited lower adhesion scores of 1 and 0.33, respectively (Figure 10g). On postoperative day 21, the adhesion severity scores were 2.33 for the control group, 1.67 for the commercial product group, 1 for the HA-GA (1 wt%)/PluF hydrogel, and 1.33 for the HA-GA (2 wt%)/PluF hydrogel. Both HA-GA/PluF hydrogels exhibited lower adhesion severity scores than the control and commercial product groups 21 d after surgery. The HA-GA/PluF hydrogels were designed to combine conformable temperature-sensitive gelation with enhanced wet-tissue retention, which may have contributed to its improved performance under experimental conditions. The effect of HA-GA concentration was more evident during the early postoperative period, with the HA-GA (2 wt%)/PluF formulation showing greater reductions in both adhesion extent and severity than the HA-GA (1 wt%)/PluF formulation on postoperative day 7. In the severe cecum–abdominal wall abrasion model, the commercial comparator showed a reduction in adhesion severity compared with the untreated control, whereas no clear reduction was observed in the adhesion extent score. These findings may reflect differences in material performance under the specific experimental conditions, including the severity of tissue injury, barrier conformity, and local material retention.

In addition, this study has several limitations related to the in vitro and in vivo experimental conditions. Postoperative adhesion formation is initiated during the early wound-healing stage, which involves fibrin deposition followed by fibroblast migration and extracellular matrix deposition when fibrinolysis is insufficient [2,4]. Accordingly, maintaining a physical barrier during this early postoperative period may play an important role in limiting contact between injured tissue surfaces and subsequent adhesion formation [7]. Although the present in vivo retention study focused on the early postoperative period, the longer-term fate and degradation behavior of HA-GA/PluF hydrogels remain to be further investigated. In addition, the in vitro flow-based retention model used in this study does not fully reproduce the complex physiological environment of the peritoneal cavity, where peritoneal fluid, proteins, cells, enzymatic activity, inflammatory mediators, and repetitive organ motion may affect hydrogel retention and erosion. Future studies incorporating more physiologically relevant fluid compositions and dynamic mechanical conditions would therefore be valuable for further evaluating hydrogel performance. Histological analysis and assessment of local inflammatory and tissue responses at the hydrogel–tissue interface would also provide additional information regarding the biological response to the material.

## 4. Conclusions

In this study, injectable thermosensitive anti-adhesive HA-GA/PluF hydrogels were developed for enhanced immobilization and retention of anti-adhesive materials to prevent postsurgical adhesion. The incorporation of HA-GA into PluF hydrogels preserved the beneficial thermosensitive behavior of PluF while enhancing its viscoelastic properties at physiological temperature. Notably, the HA-GA/PluF hydrogels showed improved in vitro and in vivo stability compared to the rapid erosion of PluF alone. In the rat cecum abrasion model, the HA-GA/PluF hydrogel effectively reduced postoperative adhesion formation, exhibiting adhesion extent and severity scores on postoperative days 7 and 21, respectively, that were lower than those in untreated control groups. Therefore, the HA-GA/PluF hydrogels exhibited reversible thermoresponsive behavior, relatively enhanced stability, prolonged retention, and effective postoperative anti-adhesion performance, highlighting their potential as promising injectable biomimetic barriers for clinical adhesion prevention.

## Data Availability

Data is contained within the article.

## Abbreviations

The following abbreviations are used in this manuscript:

HA	Hyaluronic acid
GA	Gallic acid
HA-GA	Gallic acid-conjugated hyaluronic acid
PluF	Pluronic F127
HA-GA/PluF	Gallic acid-conjugated hyaluronic acid/Pluronic F127
ADH	Adipic acid dihydrazide
EDC	1-ethyl-3-(3-dimethylaminopropyl)-carbodiimide hydrochloride
DDW	Distilled and deionized water
UV–vis	Ultraviolet–visible
PBS	Phosphate-buffered saline
LCST	Lower critical solution temperature
CGC	Critical gelation concentration
G′	Elastic modulus
G″	Viscous modulus
SD	Standard deviation
